# Serum proteomics reveals biomarkers for diagnosis, stratification, and mechanistic insights into cerebral microbleeds

**DOI:** 10.3389/fnagi.2026.1771506

**Published:** 2026-03-05

**Authors:** Wu-meng Yin, Liu-chang He, Guang-yi Han, An-ming Li, Hang-hang Zhu, Yuan Cao, Xin-li Xue, Lei Zhang, Chang-he Shi, Yu-ming Xu, Yun-chao Wang

**Affiliations:** 1 Department of Neurology, The First Affiliated Hospital of Zhengzhou University, Zhengzhou University, Zhengzhou, Henan, China; 2 NHC Key Laboratory of Prevention and Treatment of Cerebrovascular Diseases, Zhengzhou, Henan, China; 3 Clinical Systems Biology Laboratories, The First Affiliated Hospital of Zhengzhou University, Zhengzhou, Henan, China

**Keywords:** biomarkers, cerebral microbleeds (CMBs), ECM remodeling, IGF signaling, inflammation, MAPK/RAF/ERK signaling, serum proteomics

## Abstract

**Objective:**

Cerebral microbleeds (CMBs) are small vascular lesions detectable on MRI and are associated with increased stroke risk and cognitive decline. However, imaging-based diagnosis is limited by cost and accessibility. This study aimed to identify serum protein biomarkers for early CMB diagnosis and to elucidate molecular mechanisms underlying CMB subtypes.

**Methods:**

We enrolled 43 patients with MRI confirmed CMBs and 38 healthy controls. Serum proteomic profiling used high performance liquid chromatography coupled with tandem mass spectrometry. Differential protein expression and pathway enrichment analyses were performed. Biomarkers were selected using LASSO, support vector machine recursive feature elimination, and random forest algorithms. Validation employed enzyme linked immunosorbent assay (*n* = 60) and Western blotting (*n* = 8).

**Results:**

We identified 151 proteins that differed between CMB and control groups. Altered pathways involved inflammation, extracellular matrix remodeling, and lipid metabolism. Five proteins emerged as candidate biomarkers: MMP3, EFEMP1, TIMP1, UMOD, and UBA52. MMP3, EFEMP1, TIMP1, and UMOD showed robust validation performance with AUC values > 0.7, and EFEMP1 positively correlated with CMB burden. Subtype analysis distinguished lobar from deep CMBs, with RCN1, NEO1, and APLP1 effectively discriminating subtypes with AUC values > 0.8. Pathway analysis highlighted MAPK, RAF, and ERK signaling in deep CMBs and IGF signaling in lobar CMBs.

**Interpretation:**

This study presents the first comprehensive serum proteomic landscape of CMBs and identifies novel biomarkers with potential for noninvasive early diagnosis and subtype differentiation, supporting precision medicine approaches for CMB management. IGF and MAPK pathway signatures suggest mechanistic links between CMB subtypes and neurovascular aging.

## Introduction

Cerebral microbleeds (CMBs) are small haemorrhagic lesions caused by rupture of cerebral microvessels, detected using T2*-weighted gradient recalled echo (T2*-GRE) or susceptibility weighted imaging (SWI) on magnetic resonance imaging (MRI). As imaging markers of cerebral small vessel disease (CSVD), their prevalence ranges from 18 to 38% in the general population and increases with age (Vernooij et al., 2008) . Primary risk factors include advanced age, hypertension, lipid metabolism disorders, cerebral amyloid angiopathy (CAA), and specific genetic predispositions (Wang et al., 2021) . Although many patients with CMBs remain asymptomatic, studies show that CMBs increase the risk of ischaemic and haemorrhagic stroke by approximately three and five-fold, respectively, and are strongly associated with cognitive decline and dementia (Akoudad et al., 2015; Akoudad et al., 2016).

The number and anatomical distribution of CMBs have important prognostic and therapeutic implications. The Microbleed Anatomical Rating Scale (MARS) and Brain Observer Microbleed Scale (BOMBS) evaluate both their number and spatial distribution (Cordonnier et al., 2009; Gregoire et al., 2009) . Based on anatomical location, CMBs are classified as lobar or deep (including basal ganglia and infratentorial regions) (Cordonnier et al., 2009) . Lobar CMBs usually indicate underlying CAA, whereas deep CMBs are commonly associated with hypertensive small-vessel disease (Puy et al., 2021) . An increased CMB count substantially elevates bleeding risk in patients with ischaemic stroke undergoing reperfusion therapy or long-term antithrombotic treatment (Lovelock et al., 2010; Tsivgoulis et al., 2016) . Incorporating both anatomical distribution and lesion burden into clinical evaluation frameworks may therefore facilitate more accurate risk stratification and personalized treatment decisions.

Currently, CMBs diagnosis primarily relies on high-resolution MRI, particularly SWI (Wardlaw et al., 2013). However, high equipment costs and prolonged scanning limit its use for large scale screening and rapid pre-treatment assessment in acute stroke. In addition, the distinct pathological mechanisms underlying different CMB locations remain poorly understood, restricting clinicians’ ability to precisely assess risk and tailor interventions. There is thus an urgent need for a cost effective, widely accessible, quantitative alternative that enables rapid diagnosis, accurate risk evaluation, and better insight into the molecular pathogenesis of CMBs across anatomical locations.

To date, no studies have applied omics methodologies to explore the molecular features of CMBs. In this study, we used serum proteomics to identify potential biomarkers and elucidate molecular mechanisms based on lesion location. We enrolled 43 patients with MRI confirmed CMBs and 38 healthy CTRLs and collected serum for high throughput liquid chromatography–tandem mass spectrometry/mass spectrometry (LC-MS/MS) analysis. To improve detection sensitivity and proteomic resolution, serum samples were fractionated into 25 fractions by high performance liquid chromatography (HPLC) after depletion of high abundance proteins, generating a comprehensive proteomic profile. Patients were then stratified into lobar and deep subgroups for comparative proteomic analyses.

## Materials and methods

### Patient recruitment and clinical data collection

We consecutively enrolled 43 adults with CMBs admitted to the Department of Neurology, First Affiliated Hospital of Zhengzhou University, between March 2018 and February 2023. Inclusion criteria were age ≥ 55 years, total CSVD burden score ≥ 2, at least two CMB lesions, and modified Rankin Scale score ≤ 2 (Staals et al., 2014) . Healthy age matched CTRLs (*n* = 38) were recruited during the same period and had normal cranial MRI. Exclusion criteria were mixed type CMBs; history of or new large area infarction (diffusion weighted imaging lesion > 20 mm); acute cerebral or subarachnoid hemorrhage; neurodegenerative dementia; non vascular white matter disease; psychiatric disorders meeting DSM criteria; and intracranial infection, trauma, or tumor. The study protocol was approved by the Ethics Committee of the First Affiliated Hospital of Zhengzhou University, China (No. 2021-KY-1059-002).

Baseline data on demographics, medical history, smoking and alcohol use, family history of stroke, and antiplatelet/anticoagulant use during the 12 months prior to enrollment were collected at enrolment. Fasting venous blood was drawn to measure routine biochemical parameters, including lipid profile, blood glucose, and homocysteine. Neuroimaging was assessed independently by two neurologists blinded to clinical data, according to STRIVE-2 criteria (Duering et al., 2023) . Periventricular and deep white matter hyperintensities were graded using the Fazekas scale (1–3) (Fazekas et al., 1987) . CMBs were identified on SWI and graded as: 0, none; 1, 2–4 lesions; 2, 5–9 lesions; 3, ≥ 10 lesions (Biffi et al., 2010; Greenberg et al., 2009) . According to anatomical distribution, patients were categorized into deep and infratentorial CMBs (deep group, *n* = 18) and purely lobar CMBs (lobar group, *n* = 25).

### Mass spectrometry analysis

Peripheral venous blood samples were centrifuged at 3,200 rpm for 10 min at 4°C. For each serum sample, 8 μL was processed using High-Select HSA/Immunoglobulin Depletion Resin (Thermo Fisher Scientific, A36366) to remove high-abundance proteins (albumin and immunoglobulins). Following depletion, the proteins were precipitated with acetone, resuspended in 25 mM ammonium bicarbonate buffer via ultrasonication, and reduced using DTT (final concentration, 5 mmoL). Samples were then subjected to two rounds of trypsin digestion (12 h, followed by an additional 2 h). The resulting peptides were desalted using a C18 column (Waters 186002319).

Desalted peptides were dissolved in 100 μL 0.1% formic acid, and concentrations were measured with a colorimetric peptide assay. All 81 participants were processed and fractionated individually. For each individual sample, a total of 75 μg peptides was subjected to offline high-pH reversed-phase HPLC fractionation on a BEH C18 column into 25 fractions. Fractions were freeze-dried and stored at −80°C. Prior to LC–MS/MS analysis, each fraction was reconstituted in 12 μL 0.1% formic acid, and 2 μL was injected per run. Peptides were analyzed on an Easy nLC 1,200 coupled to a Q Exactive HF X Orbitrap mass spectrometer. Label free data dependent acquisition with higher energy collision dissociation was used. Raw files were processed in MaxQuant against the reviewed human UniProt database. Mass tolerances were 20 ppm for the first search and 4.5 ppm for the main search, and 20 ppm for fragments. Trypsin was specified as the enzyme with up to two missed cleavages. Variable modifications were methionine oxidation and N terminal acetylation. LFQ was performed at a peptide spectrum match and protein false discovery rate of 1%, requiring at least one unique peptide and enabling Match Between Runs.

### Proteomic data processing and analysis

Protein abundance data from the 81 samples were analyzed using MaxQuant. Further analyses were conducted in R Studio, where abundances were log2-transformed. Only proteins detected in ≥ 40% of the samples were retained. Missing values were imputed using the k-nearest neighbor algorithm, followed by normalization. Sample correlation analyses were performed to assess intra- and inter-group variations. Partial least squares-discriminant analysis (PLS-DA) was conducted using the “ropls” R package to reveal global protein expression patterns and sample clustering.

Differential protein expression analysis was conducted between the following groups: CMBs (*n* = 43) vs. CTRL (*n* = 36), Deep (*n* = 18) vs. CTRL (*n* = 36), Lobar (*n* = 25) vs. CTRL (*n* = 36), Deep (*n* = 18) vs. Lobar (*n* = 25). DEPs were identified using the “limma” R package. Proteins with a *p* < 0.05 were considered DEP, and those with *p* < 0.05 and |log2(FC)| > 0.4 were designated top DEPs (TopDEs). Spearman correlation analysis was conducted between DEPs (from CMBs vs. CTRL) and CMB severity (graded using a four-point scale) (Biffi et al., 2010); proteins with *p* < 0.05 and |ρ| > 0.3 were selected.

Pathway enrichment analysis was performed using DAVID (2021) and referenced the Gene Ontology (GO) (The Gene Ontology Consortium, 2019) and Reactome (Milacic et al., 2024) databases (significance threshold: *p* < 0.05). Gene Set Enrichment Analysis (GSEA) was also conducted using the “enrichplot” R package (Subramanian et al., 2005) focusing on the most significantly upregulated and downregulated pathways ranked by Normalized Enrichment Score (NES).

Protein co-expression networks were constructed using the “WGCNA” R package to identify key modules associated with clinical traits. A soft thresholding power of 4 was used to ensure scale-free topology fit (*R*^2^ = 0.85) with optimal network connectivity.

### Machine learning analyses

To refine candidate biomarkers, Least Absolute Shrinkage and Selection Operator (LASSO) regression (Li and Sillanpää, 2012), Support Vector Machine Recursive Feature Elimination (SVM-RFE) (Sanz et al., 2018), and Random Forest (RF) (Kong and Zhang, 2023) were applied using “glmnet,” “msvmRFE.R,” and “randomForest,” the R packages. LASSO, SVM-RFE, and RF analyses all used 10-fold cross-validation. Overlapping candidate proteins from all three algorithms were retained. Logistic regression models were constructed using the “glm” function and optimized via stepwise regression. To evaluate the consistency of proteins included in the diagnostic models, a bootstrap resampling procedure (500 repetitions) was applied to each of the three machine learning approaches. Model performance was assessed via receiver operating characteristic (ROC) curves, area under the curve (AUC), and accuracy (ACC) using the “pROC” R package (Robin et al., 2011) . Model calibration was further assessed by calibration curves generated using the “rms” package (Iasonos et al., 2008) . Clinical utility was assessed with Decision Curve Analysis (DCA) (Vickers and Elkin, 2006). A nomogram was developed using the “rms” package (Iasonos et al., 2008) to predict severe CMBs ( ≥ 10 lesions).

### Enzyme-linked immunosorbent assay and western blot validation

To validate candidate biomarkers, ELISA was performed on an independent cohort (30 CMB patients, 30 CTRL subjects). Detailed assay parameters, including kit type and sample volumes, are listed in Supplementary Table 1.

### Statistical analysis

Normally distributed data are presented as mean ± standard deviation and were analyzed using independent-sample *t*-tests. Non-normally distributed data are presented as medians with interquartile ranges [M (Q1, Q3)] and analyzed using non-parametric tests. Categorical variables are reported as frequencies and percentages [n (%)] and were compared using the chi-square or Fisher’s exact tests. All statistical analyses were conducted in SPSS version 27.0. ELISA and Western blot plotting and analysis were performed using GraphPad Prism version 8. A *p* < 0.05 was considered statistically significant.

## Results

### Serum proteomic profile and candidate biomarker identification for CMBs

#### Demographic and clinical characteristics

A total of 81 participants were enrolled, comprising 43 patients with MRI-confirmed CMBs and 38 healthy CTRLs. All subjects underwent comprehensive assessments, including medical history, MRI, laboratory tests, and cognitive evaluation within 48 h of enrolment. Table 1 summarizes the demographic and clinical characteristics of the study population. Compared to the CTRL group, patients with CMBs were older and exhibited higher rates of smoking, previous stroke, hypertension, hyperhomocysteinemia, dyslipidaemia, and CAA. Although serum TCHO, TG, and LDL-C levels were lower in the CMB group, these differences were not statistically significant.

**TABLE 1:** Demographic and clinical characteristics of CMBs patients and healthy controls.

Variable	Total number *n* = 81	CMB *n* = 43	Control *n* = 38	*P*-value
Age (year)	72.99 ± 2.92	66.72 ± 7.95	62.00 ± 5.98	0.003*
Sex (male, %)	54 (66.67)	31 (72.09)	23 (60.53)	0.270
Hypertension (n, %)	40 (49.38)	29 (67.44)	11 (28.95)	< 0.001*
Diabetes mellitus (n, %)	18 (22.22)	11 (25.58)	7 (18.42)	0.439
History of stroke (n, %)	31 (38.27)	27 (62.79)	4 (10.53)	< 0.001*
Smoking (n, %)	25 (30.86)	19 (44.19)	6 (15.79)	0.006*
Alcohol consumption (n, %)	16 (19.75)	12 (27.91)	4 (10.53)	0.05
History of cardiovascular disease (n, %)	9 (11.11)	6 (13.95)	3 (7.89)	0.387
Antiplatelet and anticoagulant agents (n, %)	25 (30.9)	15 (34.9)	10 (26.3)	0.405
Glycated hemoglobin, HbA1c (%)	5.72 ± 0.11	6.41 ± 1.33	6.27 ± 1.15	0.619
Dyslipidemia (n, %)	43 (53.09)	31 (72.09)	12 (31.58)	< 0.001*
Total cholesterol, TCHO (mmoL/L)	5.25 ± 0.55	4.01 ± 1.25	4.41 ± 0.95	0.113
Triglycerides, TG (mmoL/L)	1.82 ± 0.29	1.34 ± 0.64	1.52 ± 0.78	0.249
Low-density lipoprotein cholesterol, LDL-C (mmoL/L)	3.36 ± 0.35	2.44 ± 1.14	2.85 ± 0.86	0.074
High-density lipoprotein cholesterol, HDL-C (mmoL/L)	1.33 ± 0.09	1.15 ± 0.34	1.23 ± 0.34	0.306
Hyper-homocysteinemia (n, %)	25 (30.87)	18 (41.86)	7 (18.42)	0.023*
Cerebral amyloid angiopathy, CAA (n, %)	6 (7.41)	6 (13.95)	0 (0.00)	0.027*

*p < 0.05.

#### Serum proteomic profile in CMBs

**FIGURE 1:**
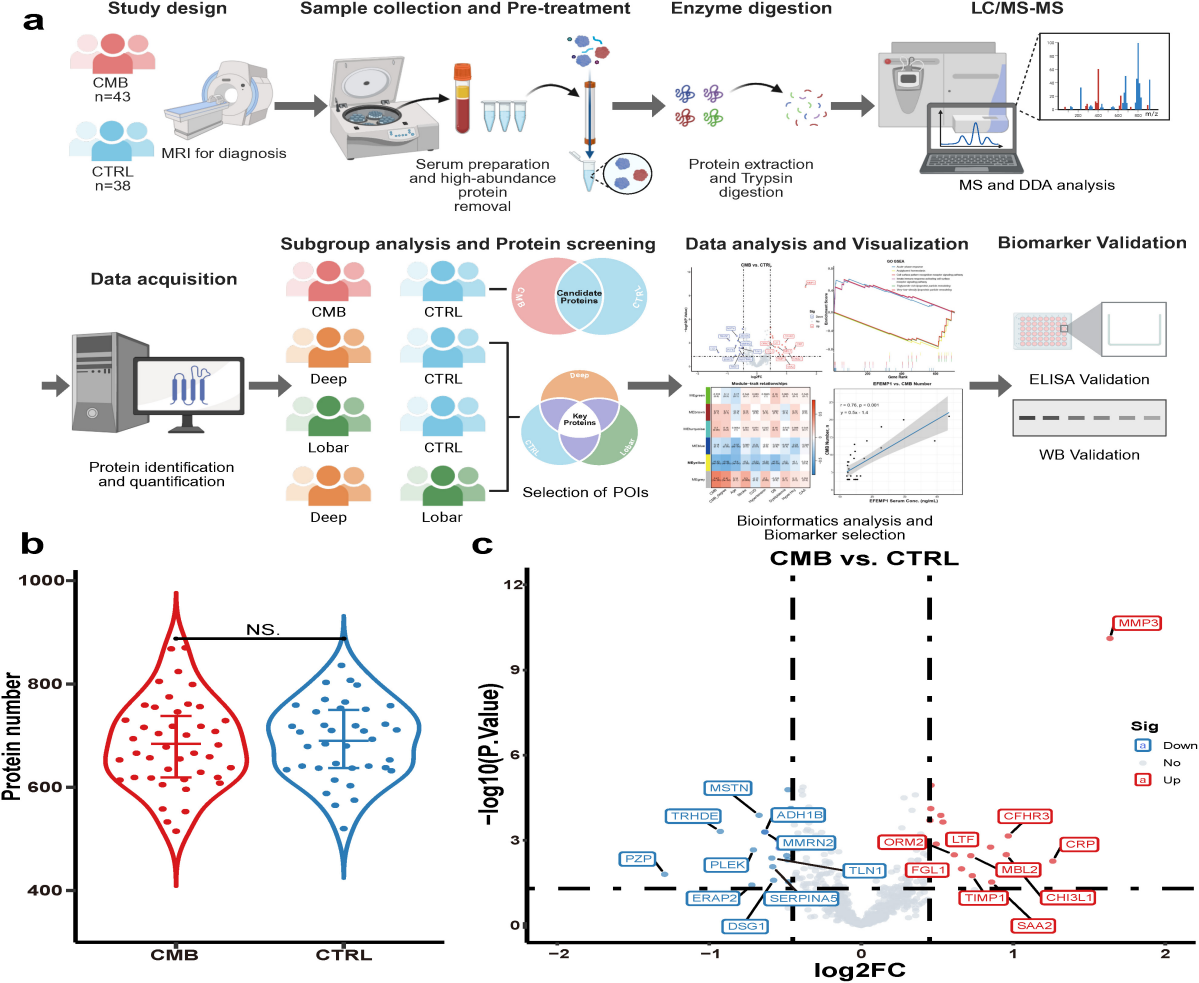
Study workflow and summary of serum proteomic profiling in CMBs. **(a)** Schematic overview of study design, proteomic analysis, subgroup comparisons, and biomarker validation (created with BioRender.com). **(b)** Violin plot showing no significant difference in identified protein counts between CMB and CTRL group (NS, not significant). **(c)** Volcano plot of significantly upregulated (red) and downregulated (blue) proteins (*p* < 0.05, |log2FC| > 0.4).

Figure 1a outlines the experimental workflow. In total, 2,062 proteins were identified across the 81 serum samples. Following the removal of high-abundance and duplicate proteins, 1,341 proteins remained for further analysis. The CMB group included 1,270 proteins, while the CTRL group included 1,261, with 1,190 shared proteins (Supplementary Figure 1a). No significant differences in the number of identified proteins were found between groups (Figure 1b). After data normalization, 714 proteins were analyzed. Two outlier samples were removed based on correlation analysis and PLS-DA (Supplementary Figures 1b,c), leaving 43 CMB and 36 CTRL samples. Differential expression analysis revealed 151 DEPs (61 upregulated, 90 downregulated), including 55 TopDEs (25 upregulated, 30 downregulated) (Figure 1c).

#### Pathway enrichment analysis

**FIGURE 2:**
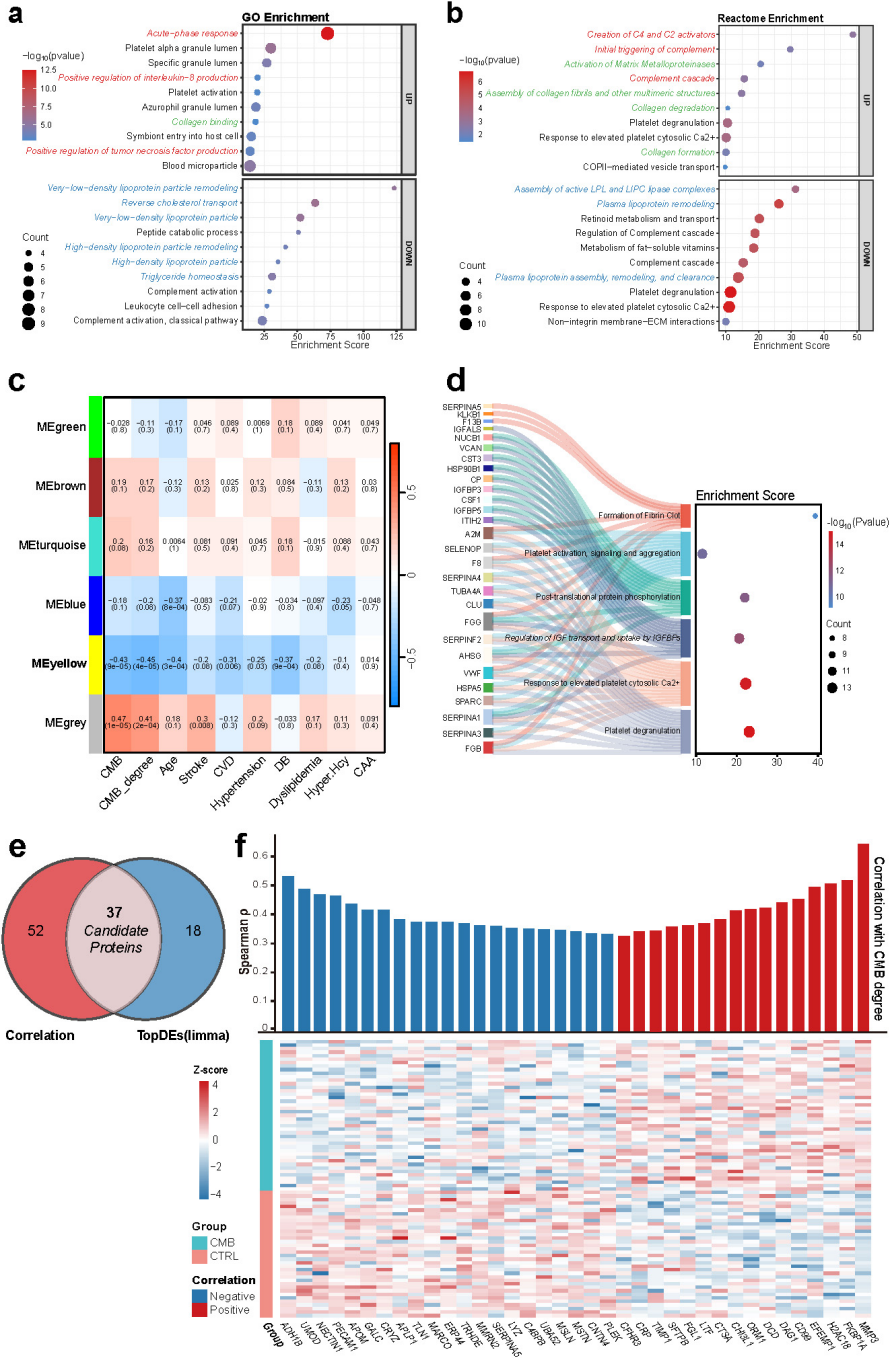
Functional enrichment, WGCNA, and candidate protein selection in CMB DEPs. **(a,b)** GO **(a)** and Reactome **(b)** enrichment of DEPs between CMB and CTRL groups. Top 10 upregulated and top 10 downregulated pathways (*p* < 0.05, ranked by enrichment score) are shown; dot size indicates the number of DEPs. Pathways related to immune/inflammatory response (red), lipid metabolism (blue) and ECM remodeling (green) are highlighted. **(c)** Heatmap of correlations (*p*-values in parentheses) between module eigengenes and clinical features in CMBs based on WGCNA. **(d)** Sankey plot of Reactome enrichment for the yellow module, showing the six most significant pathways and associated proteins; dot size indicates protein counts. **(e)** Intersection of top differentially expressed proteins (TopDEs, limma) with proteins correlated with CMB burden (Spearman |ρ| > 0.3, *p* < 0.05), yielding 37 candidates. **(f)** Bar plot and heatmap showing expression of the 37 proteins across groups and correlation with CMB grade.

GO and Reactome enrichment analyses were conducted to explore the biological functions and signaling pathways of the 151 DEPs. GO analysis showed that upregulated proteins were mainly involved in immune activation and inflammatory responses, suggesting a central role for inflammation in CMBs. Downregulated proteins were predominantly linked to lipid metabolism pathways (Figure 2a). Reactome analysis supported these findings, highlighting complement activation, innate immune response, and extracellular matrix (ECM) remodeling, including matrix metalloproteinases activation, collagen degradation, and fibril assembly, among the upregulated pathways, while lipid metabolism remained the dominant downregulated process (Figure 2b).

GSEA based on all 714 proteins further confirmed the involvement of inflammation, lipid metabolism dysregulation, and ECM. Additionally, estrogen receptor-mediated signaling emerged as a potentially protective mechanism via anti-inflammatory and vascular effects (Supplementary Figures 1d,e).

WGCNA was used to assess protein co-expression networks and their relationship with clinical features (Supplementary Figure 1f). Six co-expression modules were identified (Figure 2c). DEPs were primarily enriched in the yellow and gray modules (Supplementary Figure 1g). The yellow module showed moderate negative correlations with both the presence of CMBs (*r* = –0.43, *p* < 0.001) and severity (*r* = –0.45, *p* < 0.001), and was also negatively correlated with age, cardiovascular disease, and diabetes. Brown and cyan modules demonstrated weak and mostly non-significant positive correlations.

Reactome enrichment of the yellow module revealed six pathways, including platelet degranulation, platelet activation, fibrillar plaque formation, and IGF signaling. These processes are implicated in CMB pathogenesis and are closely associated with aging, cardiovascular conditions, and diabetes (Figure 2d).

#### Identification of diagnostic biomarkers for CMBs

Spearman correlation analysis was performed between the 714 proteins and CMB severity (Graded 0: none; 1:2–4 lesions; 2:5–9 lesions; 3: ≥ 10 lesions) (Greenberg et al., 2009) . A total of 89 proteins (*p* < 0.05, |ρ| > 0.3) were selected. Intersection with the 55 TopDEs identified 37 candidate biomarkers, accounting for 67.3% of the TopDE set (Figures 2e,f). Matrix Metallopeptidase 3 (MMP3) was the most significantly upregulated protein and showed the strongest correlation with CMB severity.

The 37 candidate proteins were further refined using LASSO, SVM-RFE, and Random Forest algorithms. LASSO yielded 26 proteins, SVM-RFE 16 proteins, and Random Forest eight proteins. The intersection of these sets produced five candidate proteins (Supplementary Figures 2a–c). In 500 bootstrap resamples, the five proteins were consistently re-selected, with selection frequencies of 0.53–1.00 (LASSO), 0.952–0.998 (SVM-RFE), and 0.40–0.99 (Random Forest). Logistic regression with bidirectional stepwise selection confirmed the final biomarkers: Uromodulin (UMOD), Ubiquitin A-52 Residue Ribosomal Protein Fusion Product 1 (UBA52), Tissue Inhibitor of Metalloproteinases 1 (TIMP1), Matrix Metallopeptidase 3 (MMP3), and EGF Containing Fibulin Extracellular Matrix Protein 1 (EFEMP1) (Supplementary Figures 2d,e).

#### Biomarker validation

**FIGURE 3:**
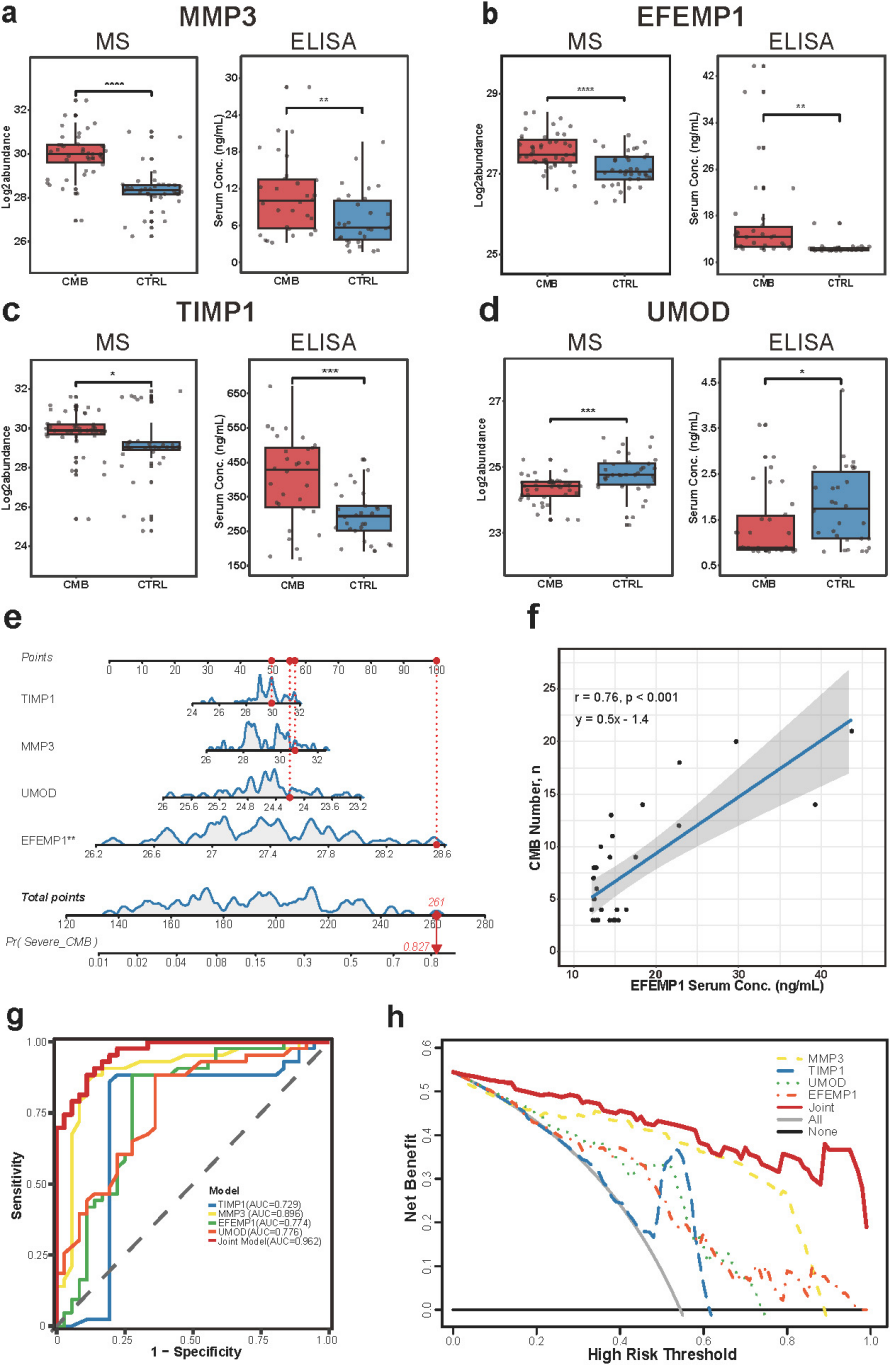
Validation and diagnostic performance of serum biomarkers for CMBs. **(a–d)** Quantitative levels of EFEMP1, MMP3, TIMP1, and UMOD in CMBs and CTRL groups, measured by mass spectrometry (left) and validated by ELISA (right). Statistical significance determined by limma (discovery) and unpaired *t*-test (validation). *p < 0.05, **p < 0.01, ***p < 0.001, ****p < 1 × 10^−4^. **(e)** Nomogram for predicting severe CMB risk (CMB number ≥ 10) based on six selected proteins; * indicates statistical significance in multivariate logistic regression. **(f)** Linear regression of serum EFEMP1 concentration (ELISA, validation cohort) and CMB number (*r* = 0.76, *p* < 0.001). **(g)** ROC curves for the four validated biomarkers and their combined model in detecting CMBs. **(h)** Decision curve analysis (DCA) comparing the net clinical benefit of individual biomarkers and the joint panel for CMB diagnosis.

To validate the diagnostic model, ELISA was performed on 30 patients with CMBs and 30 CTRLs. Serum levels of MMP1, EFEMP1, UBA52, UMOD, and TIMP1 were quantified. Significant differences were observed for MMP1, EFEMP1, TIMP1, and UMOD (Figures 3a–d and Supplementary Figure 2f).

Given that a CMB count ≥ 10 is a high-risk marker for haemorrhagic transformation after reperfusion therapy (Tsivgoulis et al., 2016), we constructed a binary logistic regression model using the four validated proteins to identify patients with severe CMB burden. A nomogram was developed to visualize predictive performance (Figure 3e). The model successfully stratified high-risk patients, generating a probability score (*p* = 0.827). EFEMP1 exhibited high inter-individual variability and a strong correlation with CMB severity, supporting its utility in rapid screening.

Further analysis showed minimal EFEMP1 variation in CTRLs but pronounced variability in patients with CMBs. This aligned with nomogram predictions and suggested EFEMP1 as a promising marker for CMB severity. Linear regression revealed a strong positive correlation between EFEMP1 levels and CMB count (*r* = 0.76, *p* < 0.001) (Figure 3f).

ROC curves showed high diagnostic performance: MMP1 (AUC = 0.896), EFEMP1 (0.774), TIMP1 (0.729), and UMOD (0.776). The four-protein model yielded an AUC of 0.962 (Figure 3g). DCA confirmed robust clinical utility for MMP1 and EFEMP1 across risk thresholds, and the combined model provided strong decision support (Figure 3h). Calibration analysis further indicated good agreement between predicted and observed probabilities for the four-protein model, with the bootstrap bias-corrected calibration curve closely tracking the ideal line (Supplementary Figure 2g).

### Protein expression profiles and identification of specific biomarkers in different CMBs locations

#### Demographic and clinical characteristics

Among the 43 patients with CMBs enrolled in this study, two experienced neurologists categorized them into deep-type (*n* = 18) and lobar-type (*n* = 25) groups based on the anatomical distribution of lesions. Patients with mixed-type CMBs were excluded due to their complex pathological background. Statistical analyses were performed to compare the demographic and clinical features of the two subgroups.

Supplementary Table 2 summarizes the clinical characteristics of the deep and lobar CMB groups, showing a significantly higher prevalence of hypertension in the deep group. Among other CSVD-related imaging features, only the number of CMBs and the PWMH burden differed significantly between the two groups.

#### Construction of serum proteomic profiles and screening of characteristic proteins for CMBs at different locations

**FIGURE 4:**
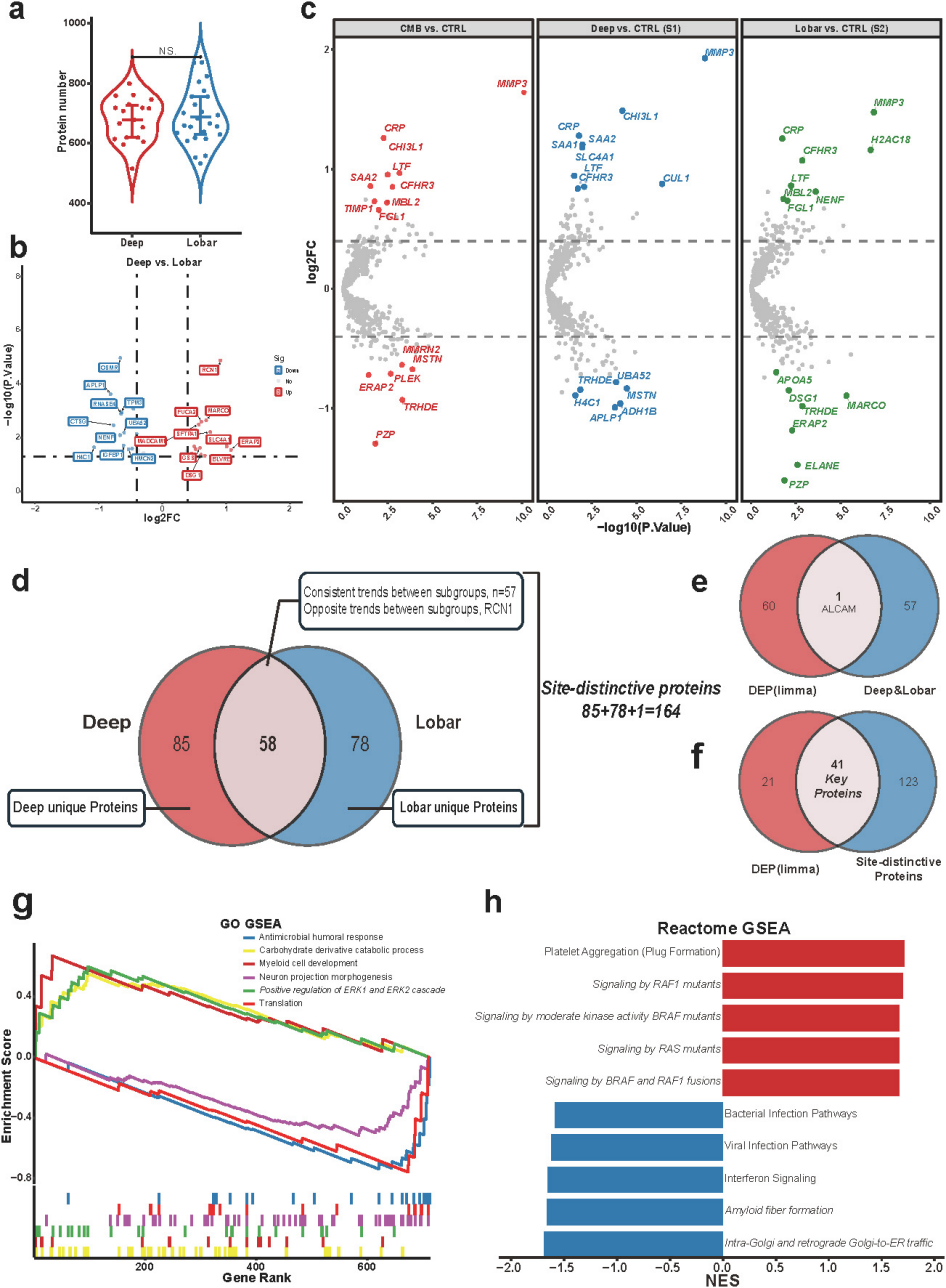
Site-specific protein identification and pathway enrichment in Deep and Lobar CMBs. **(a)**. Violin plot showing no significant difference in the total number of proteins identified between groups (NS). **(b)** Volcano plot of upregulated (red) and downregulated (blue) proteins (*p* < 0.05, |log2FC| > 0.4). **(c)** Volcano plots of DEPs in CMBs vs. CTRL, deep vs. CTRL (S1), and lobar vs. CTRL (S2), highlighting site specific expression trends. **(d)** Schematic summarizing derivation of shared proteins between S1 and S2 and selection of site distinctive proteins. **(e)** Venn diagram showing overlap between 61 DEPs (excluding RCN1) from deep vs. lobar and 57 shared proteins from S1 and S2. **(f)** Venn diagram showing the intersection between 164 site distinctive proteins and 62 DEPs, identifying 41 key proteins. **(g,h)** GSEA plots based on GO **(g)** and Reactome **(h)** for deep vs. lobar; GO shows the top three upregulated and downregulated pathways, and Reactome the top five, ranked by normalized enrichment score (NES, *p* < 0.05); selected pathways are italicized.

In the comparison between deep and lobar groups, 1,113 proteins were identified in the deep group and 1,231 in the lobar group, with no significant difference in the total protein count (Figure 4a). PLS-DA analysis revealed a distinct proteomic separation between the two groups (Supplementary Figure 3a). After normalization, 713 proteins were analyzed, and 62 DEPs were identified (27 upregulated, 35 downregulated) (Figure 4b). Given that CMB count and PWMH burden were the only significantly differing imaging features, both closely associated with lesion location (Puy et al., 2021), these DEPs likely reflect location-specific expression patterns.

To further examine site-specific protein signatures, differential expression analyses were independently conducted between the deep (*n* = 18) and lobar (*n* = 25) groups against CTRLs (*n* = 36), yielding two protein subsets (S1 and S2). MMP3 was significantly upregulated in both subsets, particularly in the deep group. In contrast, APLP1 was most prominently downregulated in the deep group, while PZP was most significantly downregulated in the lobar group (Figure 4c). The partial overlap between S1 and S2 suggests divergent underlying mechanisms for deep and lobar CMBs.

Since CSVD imaging features such as WMH, perivascular space, and lacunar infarction were minimal in CTRLs, the identified differences in S1 and S2 likely reflect contributions from both CMBs and CSVD traits. To isolate the influence of CMB location, we first intersected S1 and S2 to identify 58 shared proteins. These were categorized into two types: those with consistent trends (suggestive of shared CSVD pathology) and those with opposite trends (indicative of CMB location specificity). Only one protein, RCN1, exhibited opposite expression patterns (Figure 4d).

To verify the location-specific significance of RCN1 and eliminate the confounding influence of the other 57 shared proteins, we compared the 61 DEPs (excluding RCN1) from the deep vs. lobar comparison with the 58 shared proteins. Only one protein, ALCAM, overlapped (Figure 4e), validating our screening approach. We then combined the non-overlapping proteins from S1 and S2 with RCN1 to construct a set of 164 “specific proteins” distinguishing CMB locations (Figure 4d). The intersection of these 164 proteins with the 62 DEPs yielded 41 overlapping proteins (66.13% of the DEPs), defined as “key proteins” for CMB location and used for downstream analysis (Figure 4f).

#### Pathway enrichment analysis

GO and Reactome enrichment analyses of the 62 DEPs from the deep vs lobar comparison were first conducted (Supplementary Figures 3b,c). Due to the limited number of DEPs, enrichment specificity was modest. GO results showed that upregulated proteins in the deep group were mainly involved in lysosomal function and collagen remodeling, while downregulated proteins were related to cell adhesion and axon guidance. Reactome analysis highlighted upregulation of erythrocyte gas exchange pathways in deep CMBs and downregulation of IGF signaling and axon guidance. These findings suggest greater vascular remodeling in deep CMBs and impaired IGF signaling in lobar CMBs.

GSEA of the full 713-protein dataset revealed significant upregulation of MAPK/RAF/ERK signaling in deep CMBs, consistent with activated angiogenesis (Dong et al., 2015; Wang et al., 2022). Lobar CMBs were enriched in “Amyloid fiber formation,” pointing to elevated Aβ deposition (Figures 4g,h and Supplementary Figure 3d).

Reactome enrichment of the 41 “key proteins” showed IGF signaling regulation as the top pathway by *p*-value and NES, further linking this pathway to anatomical CMB distinctions (Supplementary Figure 3e).

#### Biomarker selection for CMBs location differentiation

Feature selection was performed on the 41 “key proteins” using LASSO regression, SVM-RFE, Random Forest, and logistic regression. LASSO selected 18 proteins, SVM-RFE selected 26, and Random Forest selected 16 (Supplementary Figures 3f–h). Eight proteins were shared across all methods: APLP1, MADCAM1, RCN1, FUCA2, RNASE4, NEO1, and MARCO (Supplementary Figure 4a). Notably, RCN1 and FUCA2 participate in IGFBP-related signaling. Logistic regression models based on AUC ranking were constructed by sequentially combining these proteins. The top four—RCN1, NEO1, APLP1, and MADCAM1—formed the optimal biomarker panel for CMB location differentiation, achieving the highest AUC with the fewest proteins (Supplementary Figures 4b–j). In 500 bootstrap resamples, all four proteins consistently ranked within the top 10 among the 41 candidates across LASSO, SVM-RFE, and Random Forest.

#### Validation of biomarkers

**FIGURE 5:**
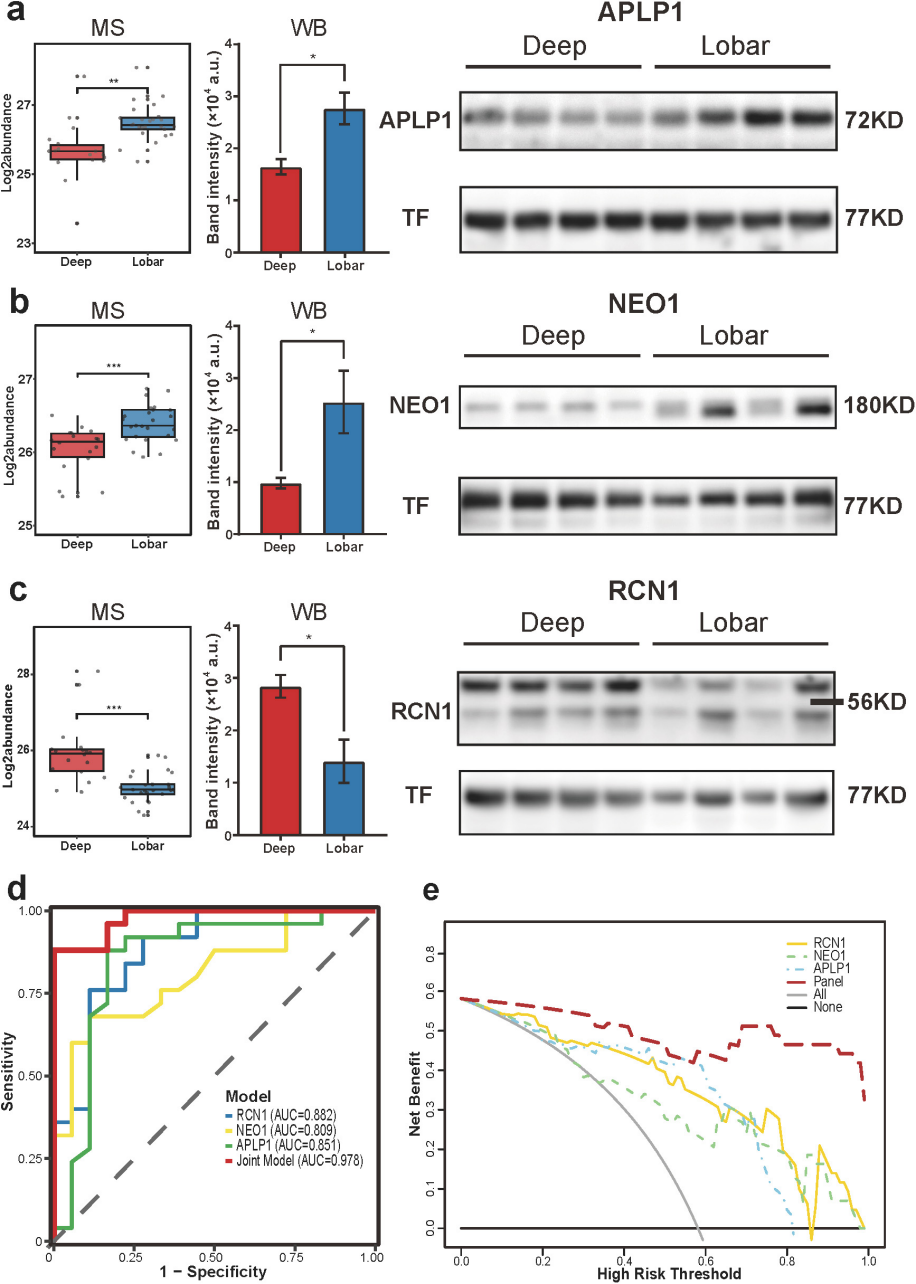
Validation of site-distinctive biomarkers for Deep and Lobar CMBs. **(a–c)** Quantitative analysis of APLP1 **(a)**, NEO1 **(b)**, and RCN1 **(c)** in Deep and Lobar CMB subgroups measured by mass spectrometry (left) and validated by Western blot (middle and right). Statistical significance determined by limma (discovery) and unpaired *t*-test (validation). WB band intensities were quantified using ImageJ. *p < 0.05, **p < 0.01, ***p < 0.001. **(d)** ROC curves for RCN1, APLP1, NEO1, and their combination for distinguishing Deep and Lobar CMBs. **(e)** Decision curve analysis (DCA) evaluating the net clinical benefit of individual and joint biomarker models for distinguishing CMB subtypes.

To validate the four-protein model, serum samples were collected from 8 CMB patients (deep, *n* = 4; lobar, *n* = 4). Expression levels of RCN1, APLP1, NEO1, and MADCAM1 were analyzed via Western blotting (Figures 5a–c). RCN1 was upregulated in deep CMBs, while APLP1 and NEO1 were downregulated in lobar CMBs, aligning with discovery cohort findings. MADCAM1 showed no significant difference (Supplementary Figure 4l). ROC curve analysis revealed strong diagnostic performance: RCN1 (AUC = 0.882), APLP1 (0.851), and NEO1 (0.809). The combined three-protein model achieved an AUC of 0.978 (Figure 5d) and further calibration analysis demonstrated good calibration, with the bootstrap bias-corrected calibration curve closely aligned with the ideal line (Supplementary Figure 4k). DCA indicated substantial net clinical benefit of the three-protein model for distinguishing CMB locations (Figure 5e). These biomarkers offer novel insights into the location-specific molecular mechanisms of CMBs and may enable more precise CMB subtyping.

## Discussion

In this study, we analyzed the serum proteomes of 43 patients with CMBs and 38 healthy controls using HPLC coupled with LC MS/MS, providing a detailed view of CMBs in relation to lesion burden and anatomical location. To our knowledge, this is the first comprehensive characterization of the serum proteomic landscape of CMBs. Enrichment analyses of DEPs highlighted key roles of inflammation, ECM remodeling, and lipid metabolic dysfunction in CMB occurrence and progression. By integrating multiple machine-learning algorithms and validating an independent cohort by ELISA, we established a diagnostic model based on MMP3, EFEMP1, TIMP1, and UMOD for rapid clinical detection of CMBs. EFEMP1 also emerged as a promising indicator of CMB burden. As baseline CMB number is crucial for guiding antithrombotic and thrombolytic decisions (Schlemm et al., 2020; Wang et al., 2014), EFEMP1 may enable rapid risk assessment of haemorrhagic complications in acute stroke.

Lesion location is a major determinant of CMB pathogenesis and prognosis. Our comparative analysis of serum proteomic signatures between deep and lobar CMBs showed that MAPK/RAF/ERK signaling abnormalities predominate in deep CMBs, whereas IGF pathway dysfunction characterizes lobar CMBs. Western blotting confirmed differential expression of RCN1, APLP1, and NEO1 between subtypes. These proteins may assist in distinguishing CMB subtypes and offer a molecular framework for studying their distinct mechanisms. Previous work indicates that lobar CMBs carry a higher risk of symptomatic intracerebral hemorrhage than deep CMBs, requiring caution in antithrombotic therapy (Akoudad et al., 2015; Wilson and Werring, 2017) . Our serum biomarker panels may support accessible CMB subtyping and mechanism driven studies.

Inflammation plays a central role in CMB pathogenesis. Both perivascular Aβ deposition and hypertension-induced angiotensin II signaling can induce vascular inflammation, characterized by astrocyte activation and proliferation surrounding affected vessels (Low et al., 2019; O’Connor and Clark, 2018) . This inflammatory cascade includes the release of NF-κB and TNF-α, activation of MMPs, and microglia recruitment, ultimately disrupting the blood–brain barrier (BBB) and promoting CMB formation (Wang et al., 2021) . In our study, serum proteomic enrichment highlighted upregulation of interleukin-8 (IL-8) and TNF-α production, and MMP activation in patients with CMBs, underscoring the inflammatory basis of CMBs and supporting inflammation as a potential therapeutic target.

ECM remodeling is another fundamental mechanism contributing to CMB pathophysiology. Chronic vascular stress, Aβ accumulation, and aging trigger reactive oxygen species production in vascular smooth muscle cells, which in turn activate MMPs and degrade matrix collagen. These changes promote fibrinoid necrosis and microaneurysm formation, raising CMB susceptibility (Toth et al., 2015; Ungvari et al., 2017; Wakisaka et al., 2010) . Our findings revealed upregulation of collagen fibril assembly and degradation pathways, along with IGF signaling enrichment in the WGCNA yellow module. These results suggest that age-related IGF-1 decline may exacerbate ECM disruption (Tarantini et al., 2017) . Thus, targeting reactive oxygen species and MMP activity or restoring IGF signaling could offer novel avenues for CMB prevention and therapy.

Although the relationship between lipid metabolism and CMBs remains complex and contentious, several studies have associated low levels of total cholesterol, LDL-C, and triglycerides with increased CMB risk (Lee et al., 2002; Wieberdink et al., 2011), a trend echoed in our cohort. A reduced cholesterol status may impair vascular wall repair and elevate microbleeding susceptibility (Gurevitz et al., 2022). In our proteomic analysis, downregulation of lipid metabolism pathways involving VLDL, LDL, HDL, and TG strongly implicated dyslipidaemia in CMB pathogenesis. Future investigations are warranted to clarify these associations and assess therapeutic implications.

Analysis of DEPs between deep and lobar subtypes revealed significant upregulation of MAPK/RAF/ERK signaling in the deep group, while the lobar group exhibited enriched Aβ formation and dysregulation of the IGF pathway, suggesting distinct subtype-specific pathogenic mechanisms.

The MAPK/RAF/ERK cascade, activated sequentially via Ras, Raf, MEK, and ERK, regulates cell proliferation, angiogenesis, and ECM degradation (Kolch, 2005) . In deep CMBs, hypertension-induced vascular injury causes cerebral hypoxia and ischaemia, leading to glial cell, endothelial cell, and smooth muscle cell activation and increased VEGF-α expression (Greenberg and Jin, 2013; Lennmyr et al., 1998) . VEGF-α, a major effector of MAPK/RAF/ERK signaling, promotes its own expression through positive feedback, driving angiogenesis (Song and Finley, 2020). However, the resulting neovessels are often immature and permeable, making them prone to rupture and hemorrhage (Hayashi et al., 2020). Clinical evidence has demonstrated elevated plasma VEGF-α levels in deep CMBs, supporting this mechanism (Ogata et al., 2019).

**FIGURE 6:**
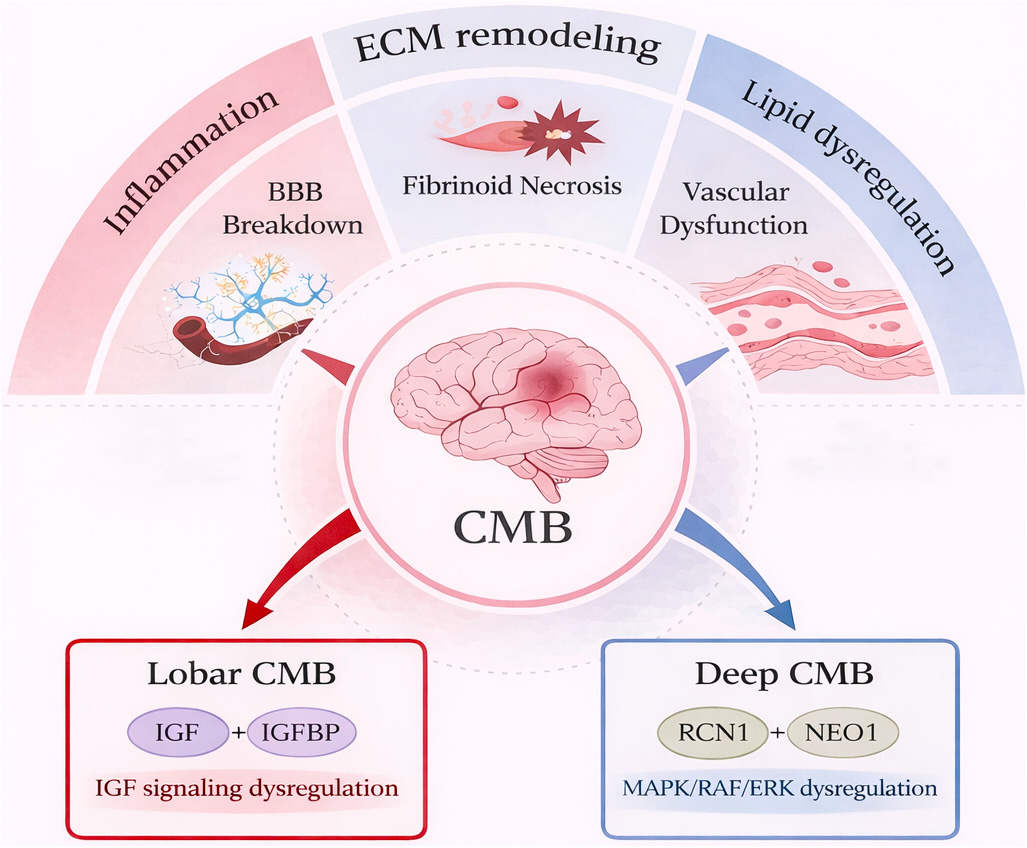
Proposed mechanisms underlying CMBs Inflammation, extracellular matrix (ECM) remodeling, and lipid metabolic dysregulation synergistically contribute to blood-brain barrier (BBB) disruption, fibrinoid necrosis, and impaired vascular repair, thereby promoting CMBs. Distinct molecular pathways differentiate CMB subtypes: Lobar CMBs are primarily associated with impaired IGF signaling, whereas Deep CMBs involve aberrant MAPK/RAF/ERK activation, and altered expression of key proteins, including RCN1 and NEO1.

The IGF signaling axis, comprising IGF-1/2, IGF-1R/2R and insulin receptors, and six binding proteins (IGFBP1–6), is vital for CNS development and Aβ homeostasis via the PI3K/Akt and MAPK/ERK pathways (Carro et al., 2002; Miao et al., 2025) . Our previous proteomic work also implicated aberrant IGF signaling in CSVD (Wang et al., 2025). IGF-1/2 downregulation or elevated IGFBP levels suppress the PI3K/Akt pathway, shift APP metabolism toward amyloidogenic processing, and accelerate Aβ accumulation (Herrero-Labrador et al., 2020; Zhang et al., 2011). This is often linked to Golgi–ER trafficking dysfunction (Wang et al., 2017). In this study, upregulation of amyloid fiber formation and intra-Golgi and retrograde ER trafficking pathways in the lobar group suggests that IGF disruption contributes to Aβ accumulation and lobar CMB pathogenesis and lobar CMB pathogenesis (Figure 6).

For CMB diagnosis, the individual AUCs for MMP3, EFEMP1, UMOD, and TIMP1 were 0.896, 0.774, 0.776, and 0.729, respectively. The combined model achieved an AUC of 0.962 and was also validated by calibration analysis, indicating excellent discriminative performance. These four proteins not only demonstrate strong diagnostic utility but also play integral roles in the underlying pathophysiology of CMBs. MMP3, a key member of the MMP family, can directly degrade the ECM and activate other MMPs (e.g., MMP2 and MMP9), thereby amplifying ECM degradation (Jiang et al., 2025) . In patients with CMBs, vascular inflammation and oxidative stress upregulate MMP3 expression, promoting ECM remodeling and BBB disruption. TIMP1, a key inhibitor of MMP, counteracts this activity and regulates angiogenesis and cell proliferation (Cabral-Pacheco et al., 2020). The dynamic interplay between MMPs and TIMP1 is essential for vascular repair and remodeling, and their ratio serves as a diagnostic and prognostic index in conditions such as Alzheimer’s disease and hypertension (Stomrud et al., 2010; Tan et al., 2007). Elevated TIMP1 in CMBs may reflect compensatory inhibition of MMP3-driven ECM degradation. EFEMP1, a core ECM protein, modulates MMPs and TIMP expression to maintain ECM homeostasis (Livingstone et al., 2020). Its elevated serum levels may represent a compensatory mechanism, promoting TIMP1 expression while inhibiting MMP3 activity, thereby mitigating ECM damage in patients with CMBs (Albig et al., 2006). UMOD, predominantly expressed in the kidney and excreted in urine, circulates at low serum levels under physiological conditions (Devuyst et al., 2017). Reduced serum UMOD is associated with increased cardiovascular risk (Steubl et al., 2020). Since renal hypertension contributes to CSVD pathogenesis (Xu et al., 2025), decreased UMOD in patients with CMBs may indicate hypertensive nephropathy. Although evidence linking UMOD to cerebrovascular diseases is limited, it remains a promising candidate for further exploration.

Location-specific CMB biomarkers identified via western blotting demonstrated strong discrimination power (RCN1: AUC = 0.882; APLP1 = 0.851; NEO1 = 0.809; combined AUC = 0.978), offering mechanistic insights into lesion localisation. RCN1, a key member of the CREC family, regulates intracellular Ca^2 +^ and suppresses ER stress-induced apoptosis (Xu et al., 2017). It also negatively modulates MAPK/RAF/ERK signaling via B-RAF inhibition (Kramann et al., 2014) . Upregulated RCN1 in deep CMBs may reflect suppression of excessive MAPK/RAF/ERK pathway activation. Chronic hypertension elevates astrocytic intracellular Ca^2 +^, impairing neurovascular unit function and causing hypoperfusion (Diaz et al., 2019); RCN1 elevation may thus indicate astrocytic compensation. Additionally, RCN1 regulates EGFR expression (Gomez et al., 2021), suggesting a role in angiogenesis and VEGF receptor modulation in deep CMBs.

NEO1, a DCC family receptor for Netrin, RGM, and BMP, is expressed in neurones (Robinson et al., 2021). Loss of astrocytic NEO1 disrupts the Netrin-NEO axis, resulting in capillary proliferation, basement membrane damage, and increased BBB permeability (Yao et al., 2020). In Moyamoya disease, NEO1 downregulation is linked to aberrant angiogenesis in VEGF-rich environments (Ren et al., 2021). Our finding of reduced NEO1 in deep CMBs supports its involvement in neovascular pathology.

APLP1, an APP family member highly expressed in neural cells, is regulated by IGF signaling and processed by α-, β-, and γ-secretases (Li and Südhof, 2004) . Unlike APP, it lacks the Aβ domain and generates intracellular domains (ALIDs) that modulate gene expression (Müller et al., 2017). APLP1 promotes non-amyloidogenic α-cleavage of APP, enhancing neuroprotective sAPPα production (Müller et al., 2017; Neumann et al., 2006). Its downregulation in lobar CMBs may reflect a compensatory mechanism to offset amyloidogenic APP processing via competitive inhibition of BACE1, thereby mitigating Aβ accumulation and slowing disease progression.

Our study had several limitations. First, although 81 participants were enrolled, the sample size remained relatively small, and the single-center, cross-sectional design may limit the generalizability of the findings. Second, while pathway enrichment and key protein identification highlighted the roles of ECM remodeling, inflammation, and lipid metabolic dysregulation in CMB pathogenesis, and revealed differences in MAPK/RAF/ERK and IGF pathways between deep and lobar subtypes, these molecular mechanisms require further experimental validation. Third, the diagnostic models developed in this study, particularly those distinguishing CMB locations, need validation in larger multicentre external cohorts to confirm their robustness and clinical utility. Lastly, patients with mixed-location CMBs were omitted, which restricts a comprehensive interpretation of the broader pathological spectrum and finer subtype stratification of CMBs.

## Conclusion

This study is the first to systematically characterize the serum proteomic landscape of patients with CMBs and elucidate the molecular mechanisms underlying different CMB subtypes. Inflammation, ECM remodeling, and lipid metabolic dysregulation were identified as central contributors to CMB onset and progression. By integrating proteomic data with multiple machine learning techniques, we identified and validated serum MMP3, EFEMP1, TIMP1, and UMOD as serum biomarkers for rapid CMB diagnosis and severity stratification. Furthermore, RCN1, NEO1, and APLP1 were revealed as subtype-specific markers capable of distinguishing lobar from deep CMBs, suggesting that the MAPK/RAF/ERK pathway is involved in deep CMBs, whereas IGF signaling is involved in the lobar subtype. Our future studies will prioritize *in vivo* investigations using animal models of CMBs to explore how modulating key subtype-specific proteins, particularly RCN1 and NEO1, influences the associated signaling pathways and clinical phenotypes. Larger cohorts incorporating more detailed location-based subtypes and longitudinal follow-up will be needed to enable finer stratification and outcome-oriented prediction. Collectively, these findings deepen our understanding of CMB pathogenesis and provide a theoretical framework for precise diagnosis and subtype-tailored management.

## Data Availability

The mass spectrometry proteomics data have been deposited to the ProteomeXchange Consortium via the iProX partner repository with the dataset identifier PXD074883.
