# Supplementary figures and images for: Serum proteomics reveals biomarkers for diagnosis, stratification, and mechanistic insights into cerebral microbleeds
Source: Front Aging Neurosci. 2026 Mar 5;18:1771506. doi: 10.3389/fnagi.2026.1771506 (PMC12999914; doi:10.3389/fnagi.2026.1771506)

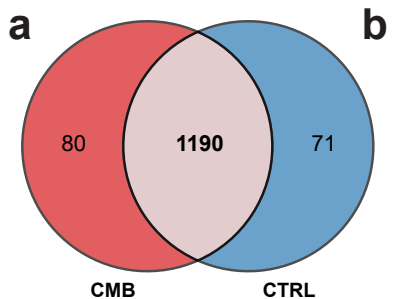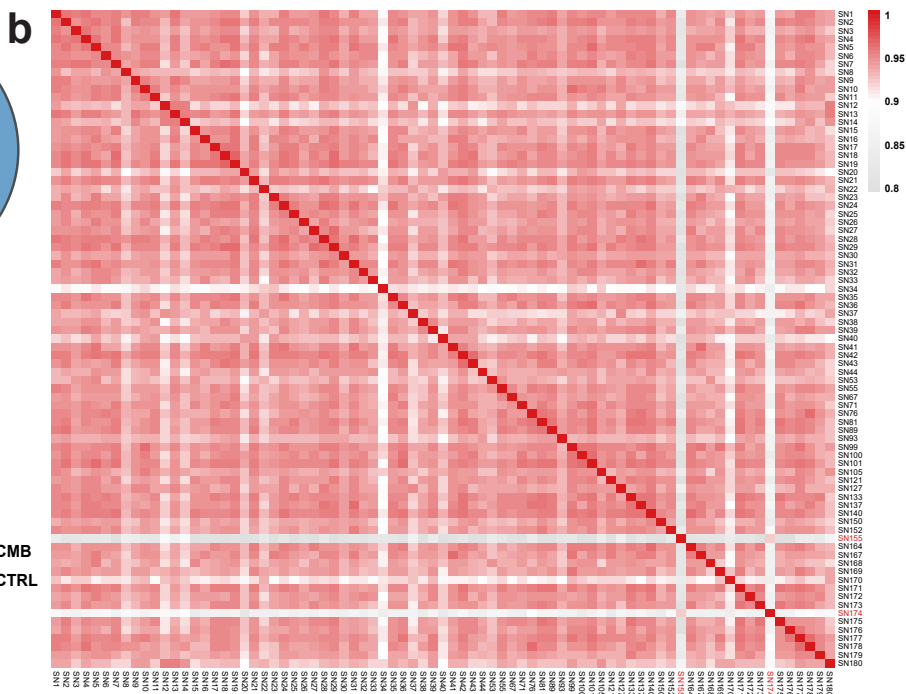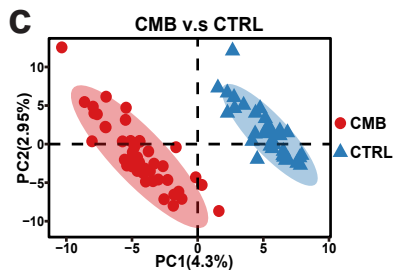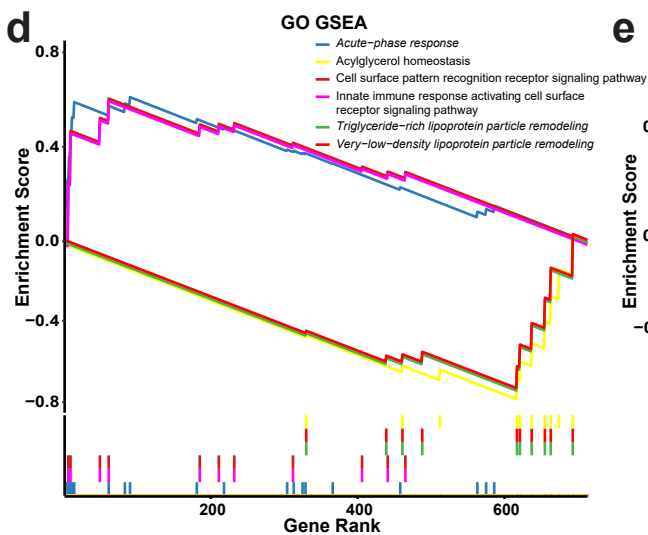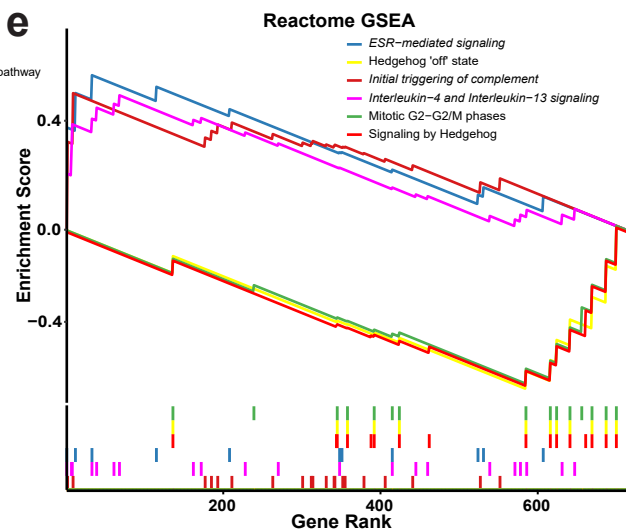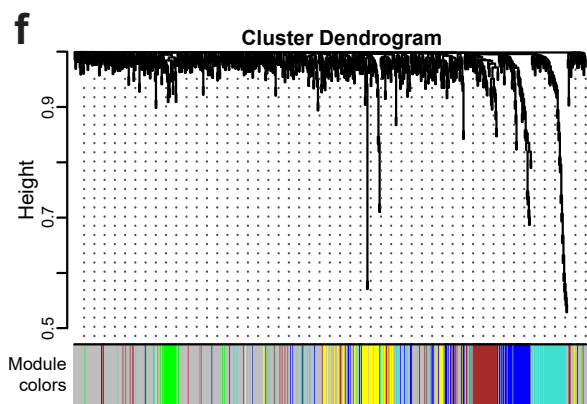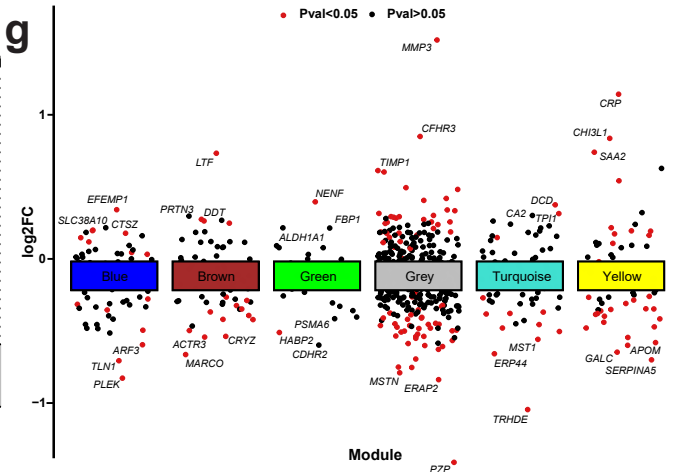

Supplement: Supplementary file 6 [file Image_1.pdf]

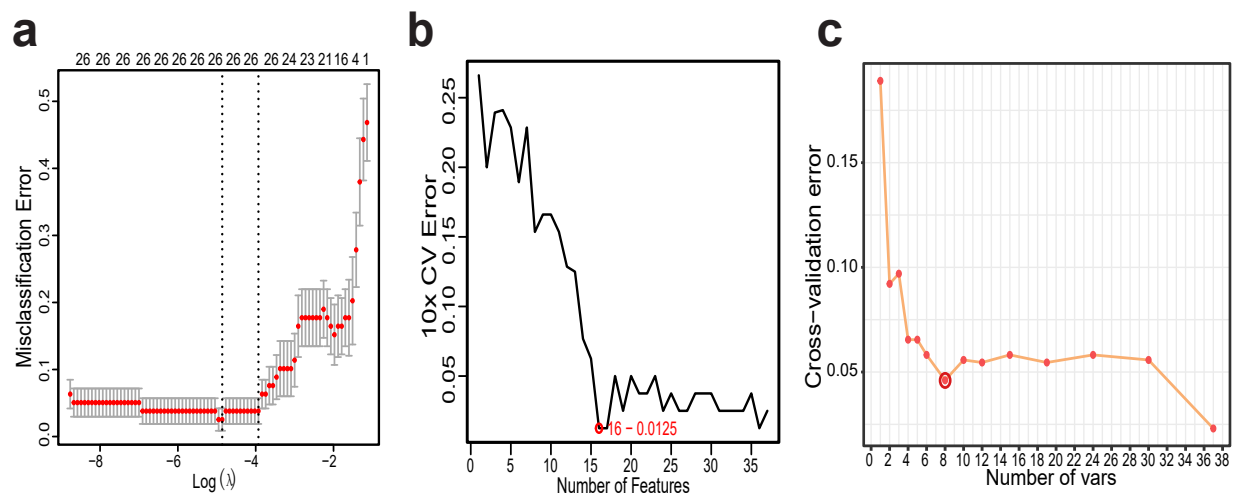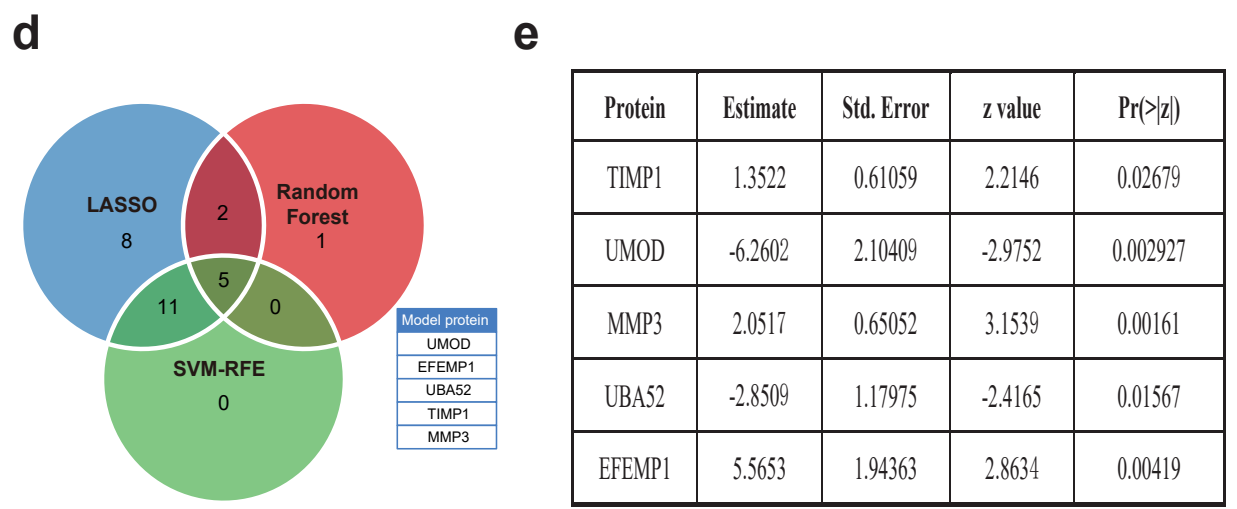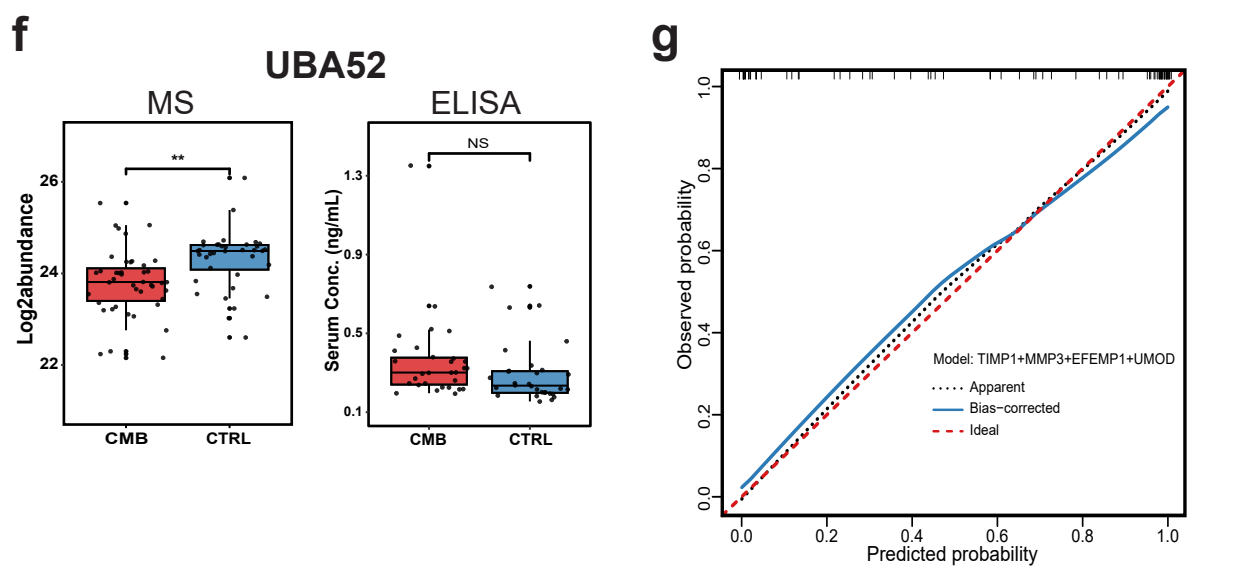

Supplement: Supplementary file 7 [file Image_2.pdf]

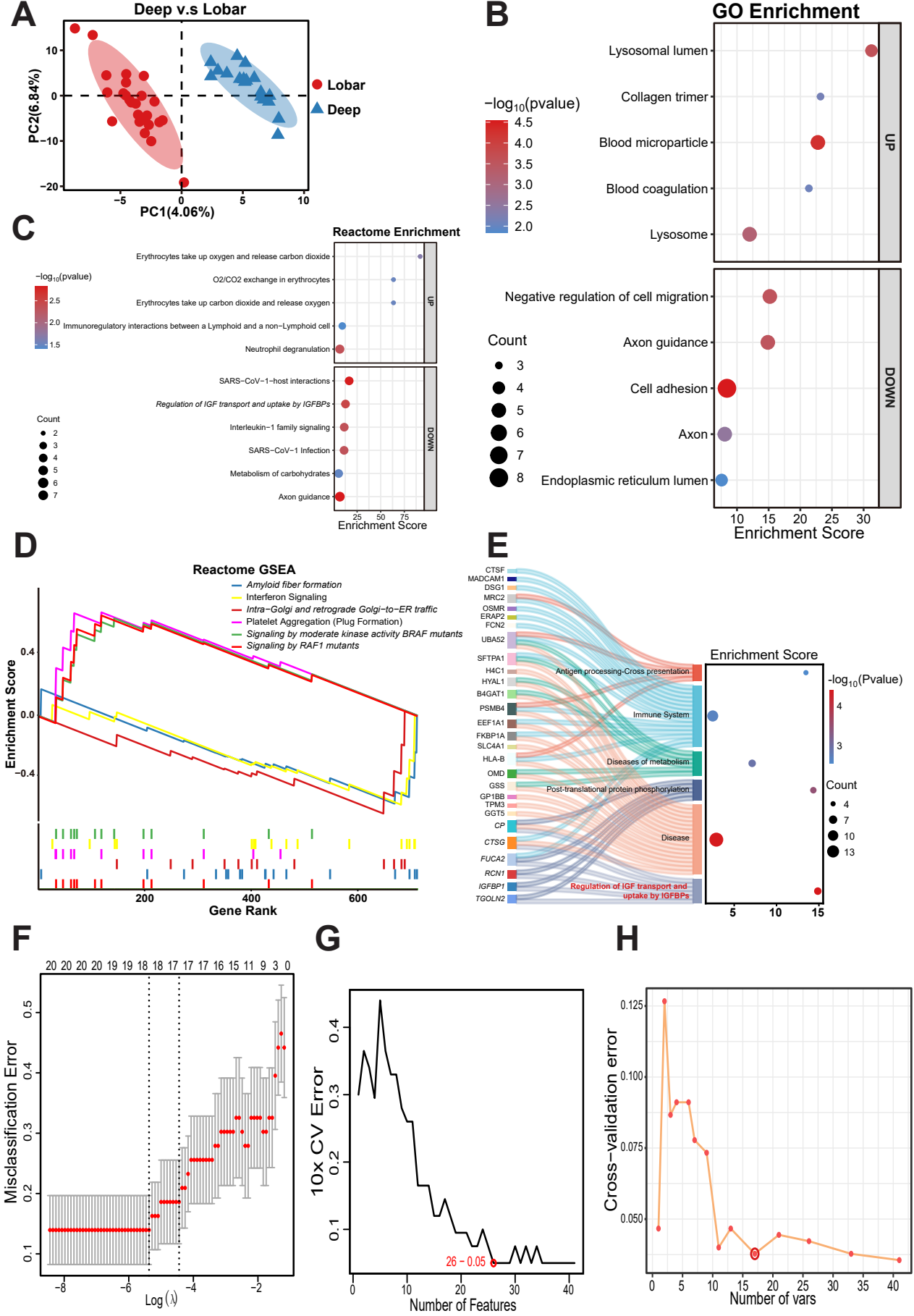

Supplement: Supplementary file 8 [file Image_3.pdf]

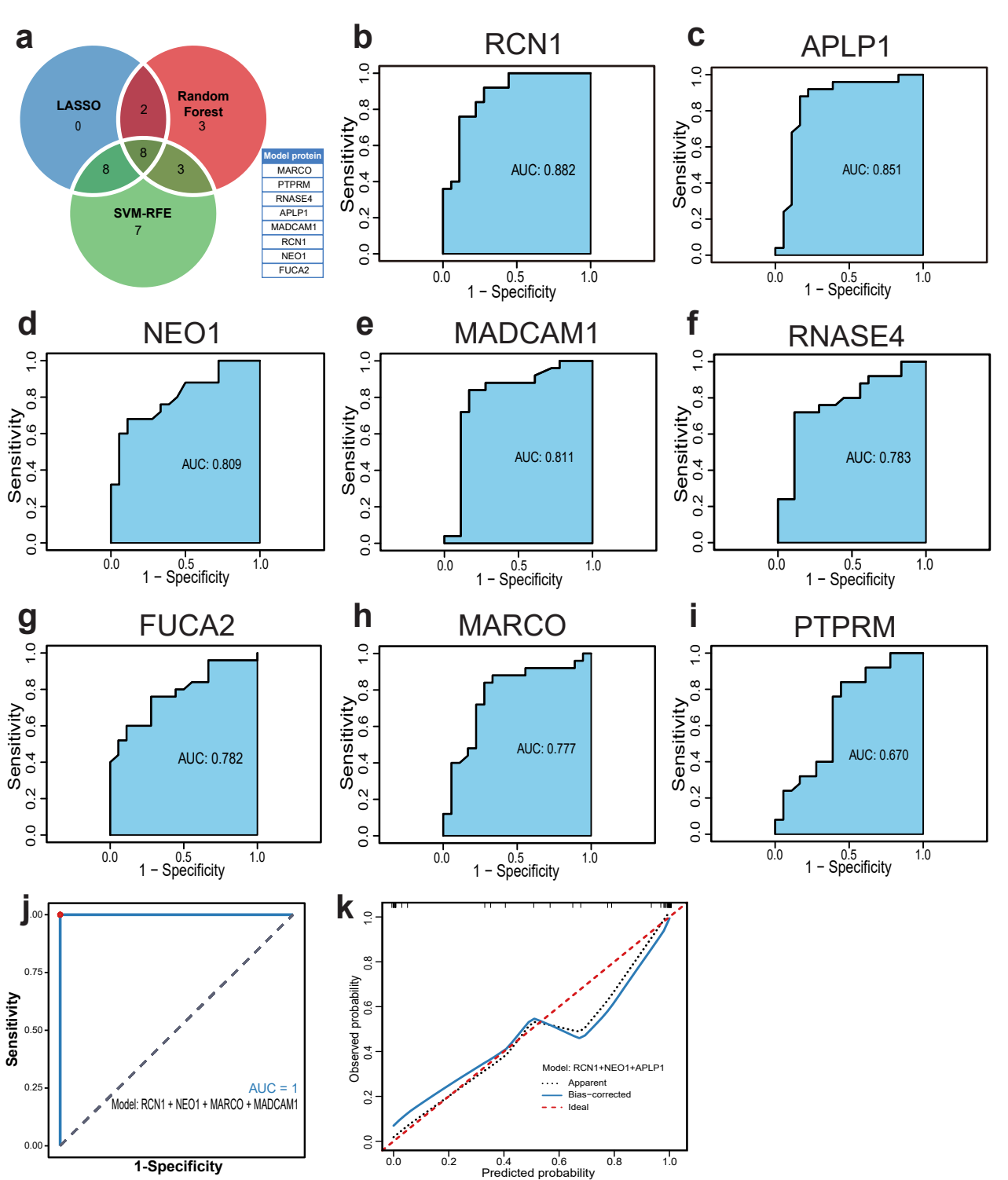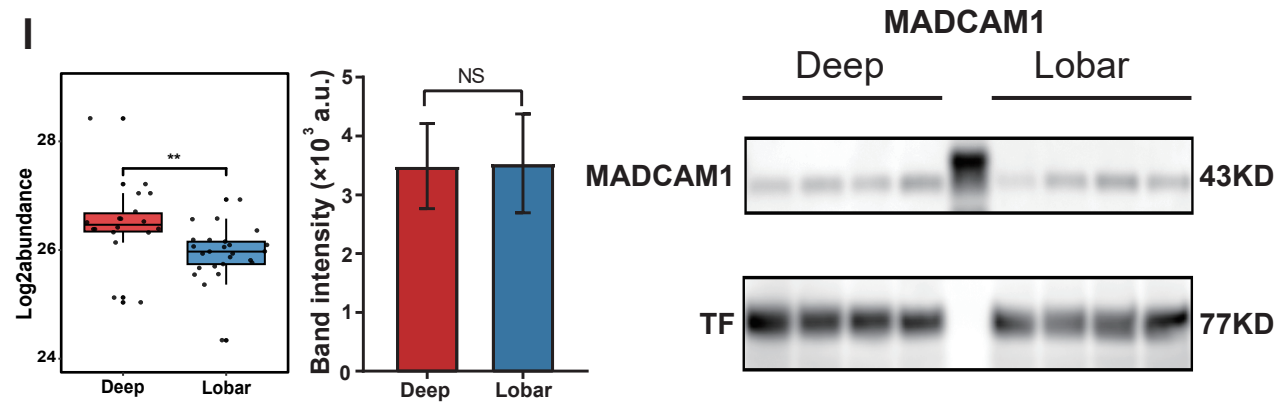

Supplement: Supplementary file 9 [file Image_4.pdf]
