# Supplementary material for: Serum proteomics reveals biomarkers for diagnosis, stratification, and mechanistic insights into cerebral microbleeds
Source: Front Aging Neurosci. 2026 Mar 5;18:1771506. doi: 10.3389/fnagi.2026.1771506 (PMC12999914; doi:10.3389/fnagi.2026.1771506)
Supplement: Supplementary file 10 [file Supplementary_file_1.docx]

| **Assay Type** | **Target Protein (Abbreviation)** | **Reagent/Antibody (Source, Catalog No.)** | **Dilution/Kit Instructions** | **Notes/Detection Instrument** |
| --- | --- | --- | --- | --- |
| ELISA | MMP3 | USCN, SEA101Hu | Per kit instructions | CMax Plus, 450 nm |
| ELISA | NECTIN1 | USCN, SEB470Hu | Per kit instructions | CMax Plus, 450 nm |
| ELISA | EFEMP1 | USCN, SEF422Hu | Per kit instructions | CMax Plus, 450 nm |
| ELISA | UMOD | USCN, SEG918Hu | Per kit instructions | CMax Plus, 450 nm |
| ELISA | UBA52 | USCN, SEE592Hu | Per kit instructions | CMax Plus, 450 nm |
| ELISA | TIMP1 | USCN, SEA552Hu | Per kit instructions | CMax Plus, 450 nm |
| WB | RCN1 | Rabbit anti-RCN1 (Invitrogen, PA5-64004) | 1:2000 | HRP-Goat anti-rabbit, 1:10000 |
| WB | NEO1 | Rabbit anti-NEO1 (Proteintech 20246-1-AP) | 1:5000 | HRP-Goat anti-rabbit, 1:10000 |
| WB | APLP1 | Rabbit anti-APLP1 (Proteintech 12305-2-AP) | 1:1000 | HRP-Goat anti-rabbit, 1:10000 |
| WB | MADCAM1 | Rabbit anti-MADCAM1 (Proteintech 21917-1-AP) | 1:1000 | HRP-Goat anti-rabbit, 1:10000 |
| WB | TF (control) | Mouse anti-TF (Proteintech 66171-1-Ig) | 1:5000 | HRP-Goat anti-mouse, 1:10000 |
| WB | Marker | Solarbio PR1960 | - | - |

**Supplementary Table 1: Western-blot and ELSIA reagents.**

**Table notes:** MMP3, Matrix Metallopeptidase 3; NECTIN1, Nectin Cell Adhesion Molecule 1; EFEMP1, EGF Containing Fibulin Extracellular Matrix Protein 1; UMOD, Uromodulin; UBA52, Ubiquitin A-52 Residue Ribosomal Protein Fusion Product 1; TIMP1, Tissue Inhibitor of Metalloproteinases 1; RCN1, Reticulocalbin-1; NEO1, Neogenin-1; APLP1, Amyloid Beta Precursor-Like Protein 1; MADCAM1, Mucosal Vascular Addressin Cell Adhesion Molecule 1; TF, Transferrin (internal control).

All ELISA assays were performed according to the manufacturer’s instructions.

For Western blot, proteins were transferred to PVDF membranes (Millipore, Immobilon-FL, IPFL00010) and detected using ECL substrate (Coolaber, SL1352). Bands were visualized with the Amersham Imager 680 imaging system, and quantification was performed with Image J software.
