# Supplementary material for: Serum proteomics reveals biomarkers for diagnosis, stratification, and mechanistic insights into cerebral microbleeds
Source: Front Aging Neurosci. 2026 Mar 5;18:1771506. doi: 10.3389/fnagi.2026.1771506 (PMC12999914; doi:10.3389/fnagi.2026.1771506)
Supplement: Supplementary file 11 [file Supplementary_file_2.docx]

| Variable | Total number  n=43 | Deep  n=18 | Lobar  n=25 | p value |
| --- | --- | --- | --- | --- |
| Age (year) | 66±7.95 | 65.50±5.89 | 67.60±9.17 | 0.399 |
| Sex (male, %) | 31(72.09) | 12(66.67) | 19(76.00) | 0.501 |
| Hypertension (n, %) | 29(67.44) | 16(88.89) | 13(52.00) | 0.011* |
| Diabetes mellitus (n, %) | 11(25.58) | 3(16.67) | 8(32.00) | 0.309 |
| History of stroke (n, %) | 27(62.79) | 9(50.00) | 18(72.00) | 0.141 |
| Smoking (n, %) | 19(44.19) | 7(38.89) | 12(48.00) | 0.553 |
| Alcohol consumption (n, %) | 12 | 6(33.33) | 6(24) | 0.501 |
| History of cardiovascular disease (n, %) | 6(13.95) | 2(11.11) | 4(16.00) | 0.648 |
| Glycated hemoglobin, HbA1c (%) | 6.42±1.33 | 6.37±1.10 | 6.45±1.51 | 0.839 |
| Total Cholesterol, TCHO (mmol/L) | 4.01±1.25 | 3.99±1.10 | 4.02±1.37 | 0.921 |
| Triglycerides, TG (mmol/L) | 1.34±0.64 | 1.41±0.69 | 1.29±0.61 | 0.546 |
| Low-density Lipoprotein cholesterol, LDL-C (mmol/L) | 2.44±1.15 | 2.38±0.96 | 2.49±1.28 | 0.759 |
| High-density Lipoprotein cholesterol, HDL-C (mmol/L) | 1.16±0.34 | 1.18±0.35 | 1.14±0.34 | 0.768 |
| Hyper-Homocysteinemia (n, %) | 18(41.86) | 7(38.89) | 11(44.00) | 0.738 |
| Cerebral amyloid angiopathy,  CAA (n, %) | 6(13.95) | 0(0.00) | 6(24.00) | 0.032* |
| CMB number, n | 9±7 | 6±2 | 12±8 | <0.001* |
| LI number, n | 3±3 | 3±3 | 3±3 | 0.908 |
| BG_PVS number, n | 13±5 | 12±5 | 13±4 | 0.533 |
| CSO_PVS number, n | 7±6 | 5±5 | 7±7 | 0.424 |
| DWMH degree, grade | 2(1,3) | 2(1,3) | 2(1,3) | 0.805 |
| PWMH degree, grade | 2(2,2) | 2(2,2) | 2(2,3) | 0.031* |
| Fazekas degree, grade | 2(2,3) | 2(2,3) | 2(2,3) | 0.772 |

**Supplementary Table 2: Demographic and Clinical Characteristics of Deep CMB Patients and Lobar CMB Patients.**

* p < 0.05
